# A Machine Learning Model for Predicting Depression in Moroccan Rheumatoid Arthritis Patients

**DOI:** 10.7759/cureus.80723

**Published:** 2025-03-17

**Authors:** Imad Chakri, Noura Qarmiche, Mohammed Omari, Nabil Tachfouti, Samira EL Fakir, Nada Otmani

**Affiliations:** 1 Medical Informatics, Laboratory of Epidemiology, Biostatistics and Health Information Processing, Agadir, MAR; 2 Biostatistics-Informatics Unit, Department of Epidemiology, Clinical Research and Community Health, Faculty of Medicine, Pharmacy and Dentistry, Sidi Mohamed Ben Abdellah University, Fez, MAR; 3 Laboratory of Epidemiology, Clinical Research and Community Health, Faculty of Medicine, Pharmacy and Dentistry, Sidi Mohamed Ben Abdellah University, Fez, MAR; 4 Department of Epidemiology, Clinical Research and Community Health, Faculty of Medicine, Pharmacy and Dentistry, Sidi Mohamed Ben Abdellah University, Fez, MAR

**Keywords:** artificial intelligence in medicine, depression prevention, machine learning (ml), rheumatoid arthriitis, support vector machine (svm)

## Abstract

Background and objective

Rheumatoid arthritis (RA) is a chronic inflammatory condition that significantly impacts the quality of life. Depression in RA exacerbates pain and reduces the likelihood of remission. Predicting depression in RA is often neglected due to time and resource constraints. Hence, this study aimed to develop a machine learning (ML) model for predicting depression in RA patients.

Methodology

We included 112 RA patients from CHU Hassan II, Fez, Morocco. Depression was assessed using the Hospital Anxiety and Depression Scale (HADS) scale, and clinical data were extracted from medical records. Twelve features were used to develop five ML models: support vector machine (SVM), random forest (RF), decision tree (DT), logistic regression (LR), and gradient boosting classifier (GBC). Data preprocessing involved managing missing values, normalizing data, and encoding variables. Model performance was evaluated using accuracy, precision, recall, F1 score, and area under the curve (AUC).

Results

The ML-based feature selection method showed the optimal performance. The LR model performed best in predicting depression, with 76.5% accuracy, 72.2% precision, 81.2% recall, an F1 score of 0.765, and an area under the receiver operating characteristic curve (ROC AUC) of 0.767.

Conclusions

Our study highlights the significance of ML models in predicting depression in RA patients. The selected features and the LR model showed promising performance. Further research is required to validate these results and develop more advanced models. Utilizing such tools could significantly impact the management of RA patients by identifying those at risk of depression and providing appropriate psychological support.

## Introduction

Rheumatoid arthritis (RA) is a chronic inflammatory disease affecting the joints and other body systems [1]. It is one of the most prevalent types of arthritis worldwide, impacting millions of individuals. The World Health Organization reports a global prevalence of approximately 1% for RA [2,3]. In Morocco, a study revealed a prevalence of around 0.5%, consistent with other low- and middle-income countries [4,5].

Patients with RA often experience psychiatric comorbidities, such as depression, anxiety, and insomnia, significantly impacting their quality of life. The prevalence of depression in RA patients is estimated to be approximately 16-20%, twice as high as the general population [6]. Several factors contribute to depression in these patients, including chronic pain, physical limitations, declining health, medication side effects, and psychosocial factors [7]. However, diagnosing and detecting depression in RA patients poses challenges as depression symptoms can overlap with the disease, and patients may be reluctant to disclose their depressive symptoms to physicians. Additionally, time and resource constraints may also hinder comprehensive psychiatric assessments for all RA patients, leading to delays in treatment and worsening mental health.

Machine learning (ML) models offer promising opportunities for disease prediction and diagnosis by analyzing large datasets to identify patterns and relationships. These models have been utilized to predict treatment outcomes, diagnose diseases, and identify risk factors within health data [8,9]. ML models have been applied in RA as well. For example, a treatment response prediction model for RA patients used ML and logistic regression (LR) with symptoms, medical history, and biological marker data [10]. Nevertheless, no specific ML models have been developed so far to predict depression in patients with RA, highlighting the need for developing such a model. The objective of this work is to develop an ML model to predict the future risk of developing depression in patients with RA.

## Materials and methods

Data source

A cross-sectional study was conducted in the Rheumatology Department at CHU Hassan II in FEZ, Morocco in September 2019. The study included a total of 112 patients diagnosed with RA for a minimum of six months. Patients with other rheumatic conditions or pre-existing psychiatric disorders before the onset of RA were excluded from the study. Data collection was performed via an anonymous face-to-face questionnaire. Clinical data were extracted from electronic medical records. The study was conducted in accordance with Good Clinical Practice (GCP) guidelines and the principles of the Declaration of Helsinki [11].

Included Variables

The collected data encompassed several categories:

Target variable for predictive modeling: The primary outcome variable was the presence of depression in patients. Depression was measured using the Hospital Anxiety and Depression Scale (HADS) [12]. The HADS questionnaire is a self-assessment scale consisting of 14 items: seven for anxiety and seven for depressive symptoms. Each item is scored from 0 to 3 based on the intensity of symptoms during the past week. A cutoff score of 8 or more was used to determine the presence of depression [13-15].

Input Variables

Table 1 presents the input variables utilized in constructing our model. The variables are grouped into four categories: sociodemographic, clinical, biological, and treatment-related factors.

**Table 1:** Variables included in the study

Variables	Details
Sociodemographic variables	Age, gender, habitat, marital status, residential status, education level, employment status, social coverage, income, smoking status
Clinical variables	Disease duration, comorbidities, joint index, synovial index, presence of deformities
Biological data	Erythrocyte sedimentation rate (ESR), c-reactive protein (CRP), rheumatoid factor (RF), anti-citrullinated peptide antibodies (ACPA), Health Assessment Questionnaire-Disability Index (HAQ-DI), Rheumatoid Arthritis Disease Activity Score (DAS28-CRP, DAS28-ESR), number of hospitalizations per year, joint manifestations
Treatments	Corticosteroids, nonsteroidal anti-inflammatory drugs (NSAIDs), analgesics, methotrexate, sulfasalazine, leflunomide, infliximab, adalimumab, etanercept, rituximab, tocilizumab, golimumab, route of methotrexate administration

Data preprocessing

Handling Missing Data

Missing data can adversely affect the performance of ML models. To address this, numerical variables with missing values were replaced with the mean of existing values for that variable, while categorical variables were replaced with the most frequent value.

Normalization of Numerical Variables

Numerical variables often have different value ranges, which can impact the performance of certain ML algorithms. To mitigate this issue, numerical variables were normalized using the minMaxScaler method, transforming the values to a range between 0 and 1.

Encoding Categorical Variables

ML algorithms require the numerical representation of categorical variables. Hence, categorical variables were encoded using the one-hot encoding method. This approach created binary variables for each category, indicating the presence or absence of that category in an observation.

Feature selection

Feature selection is a critical step in ML model development to identify the most relevant variables for predicting the target variable while minimizing overfitting [16]. Feature selection was performed using filtering and wrapper methods.

Filtering Method

The filtering method assessed the correlation between each feature and the target variable and retained the most correlated features [16,17]. Three filtering techniques were employed: correlation score, variance, and mutual information.

Correlation score (threshold ≥0.1): The first filtering technique used was the correlation score. This method involves calculating the correlation coefficient between each feature and the target variable. The correlation coefficient quantifies the strength and direction of the linear relationship between two variables. In our study, we aimed to retain features that exhibited a correlation score greater than or equal to 0.1 with the target variable.

Wrapper Method

The wrapper method involved using a model-based feature selection technique to evaluate different feature combinations and select the subset of features that improved the model's performance. Recursive feature elimination (RFE) was utilized to progressively eliminate less important features using a prediction model [18].

Model development

In the model development phase, we compared various ML algorithms for predicting depression in patients with RA. Firstly, we utilized the LR model, a statistical modeling technique that predicts a binary variable (yes or no) using a linear combination of predictive variables. We used this model to predict the presence or absence of depression in patients based on their clinical characteristics. LR is widely used in machine learning [19]. Secondly, we employed the support vector classifier (SVC) [support vector machine (SVM)] model, a powerful supervised learning algorithm that aims to find the best possible separation between different data groups by creating decision boundaries that maximize the margin between different classes. We used this model to predict the presence or absence of depression in patients with rheumatoid arthritis [19]. Thirdly, we employed the decision tree (DT) model, a non-parametric supervised learning algorithm used for classification and regression tasks. The main objective of this algorithm is to create a decision tree that facilitates decision-making by following a top-down tree path and answering questions about input variables [19]. We used the Scikit-Learn library to train our DT model and tuned hyperparameters using a 10-fold cross-validation method to improve its performance. The adjusted hyperparameters included the minimum number of samples required to split a node and the maximum depth of the tree. Lastly, to enhance accuracy and prevent overfitting, we adopted the random forest (RF) classifier model, a supervised learning algorithm that builds multiple decision trees and combines them to improve accuracy. Each decision tree is trained on a random sample of the training data.

Optimizing model performance: hyperparameter tuning with GridsearchCV

To optimize our model performance, we implemented hyperparameter tuning techniques using GridsearchCV. Hyperparameters are critical parameters that influence the behavior and performance of ML algorithms. GridsearchCV, a widely recognized method in ML optimization [19], systematically explores a predefined hyperparameter grid. Through cross-validation, each combination is evaluated to identify the optimal configuration maximizing performance metrics [20]. This exhaustive search minimizes overfitting risk and enhances model generalization [19]. By fine-tuning hyperparameters, our methodology aims to improve predictive accuracy and efficacy, ensuring applicability in real-world scenarios.

Performance and evaluation

To evaluate the model's performance in predicting depression, several metrics were used:

Precision: The number of correct positive predictions out of total positive predictions.

Recall (sensitivity): The proportion of true positive predictions among all positive instances.

Accuracy: The proportion of correct predictions (both true positives and true negatives) out of the total predictions. It measures the overall correctness of the model's predictions.

$\text{Accuracy} = \frac{\text{True Positives} + \text{True Negatives}}{\text{Total Predictions}}$

Specificity: The proportion of true negative predictions among all negative instances. It measures the model's ability to correctly identify non-depressed individuals.

$\text{Specificity} = \frac{\text{True Negatives}}{\text{True Negatives} + \text{False Positives}}$

Negative predictive value (NPV): The proportion of true negative predictions among all predictions made by the model for negative instances. NPV quantifies how well the model can correctly predict non-depressed cases.

$\text{NPV}= \frac{\text{True Negatives}}{\text{True Negatives} + \text{False Negatives}}$

F1 score: the harmonic mean of precision and recall, combining both measures: $F_1\text{-score} = 2 \times \frac{\text{Precision} \times \text{Recall}}{\text{Precision} + \text{Recall}}$

Area under the receiver operating characteristic curve (ROC AUC): This indicates the model's ability to distinguish between classes.

These metrics (formula in Table 2) were computed for each model using a test set, and ROC curves were plotted. The evaluation helped identify the most effective model for predicting depression in patients with RA.

**Table 2:** Confusion matrix with formula for various metrics The table presents a confusion matrix for a tested model, including true positives (TP), false positives (FP), true negatives (TN), and false negatives (FN)

Actual class		Tested model		Metrics
		Predicted positive	Predicted negative	
Real data	Positive	True positive (TP)	False negative (FN)	$\text{Recall} = \frac{\text{TP}}{\text{TP} + \text{FN}}$
	Negative	False positive (FP)	True negative (TN)	$\text{Specificity} = \frac{\text{TN}}{\text{TN} + \text{FP}}$
		$\text{Precision} = \frac{\text{TP}}{\text{TP} + \text{FP}}$	$\text{NPV} = \frac{\text{TN}}{\text{TN} + \text{FN}}$	$\text{Accuracy} = \frac{\text{TP} + \text{TN}}{\text{TP} + \text{TN} + \text{FP} + \text{FN}}$

## Results

Depression

The prevalence of depression, assessed using the HADS score with a threshold of 8 or more, was 48.21% in our sample.

Development of the machine learning model for predicting depression

Data Preprocessing

**Figure 1:**
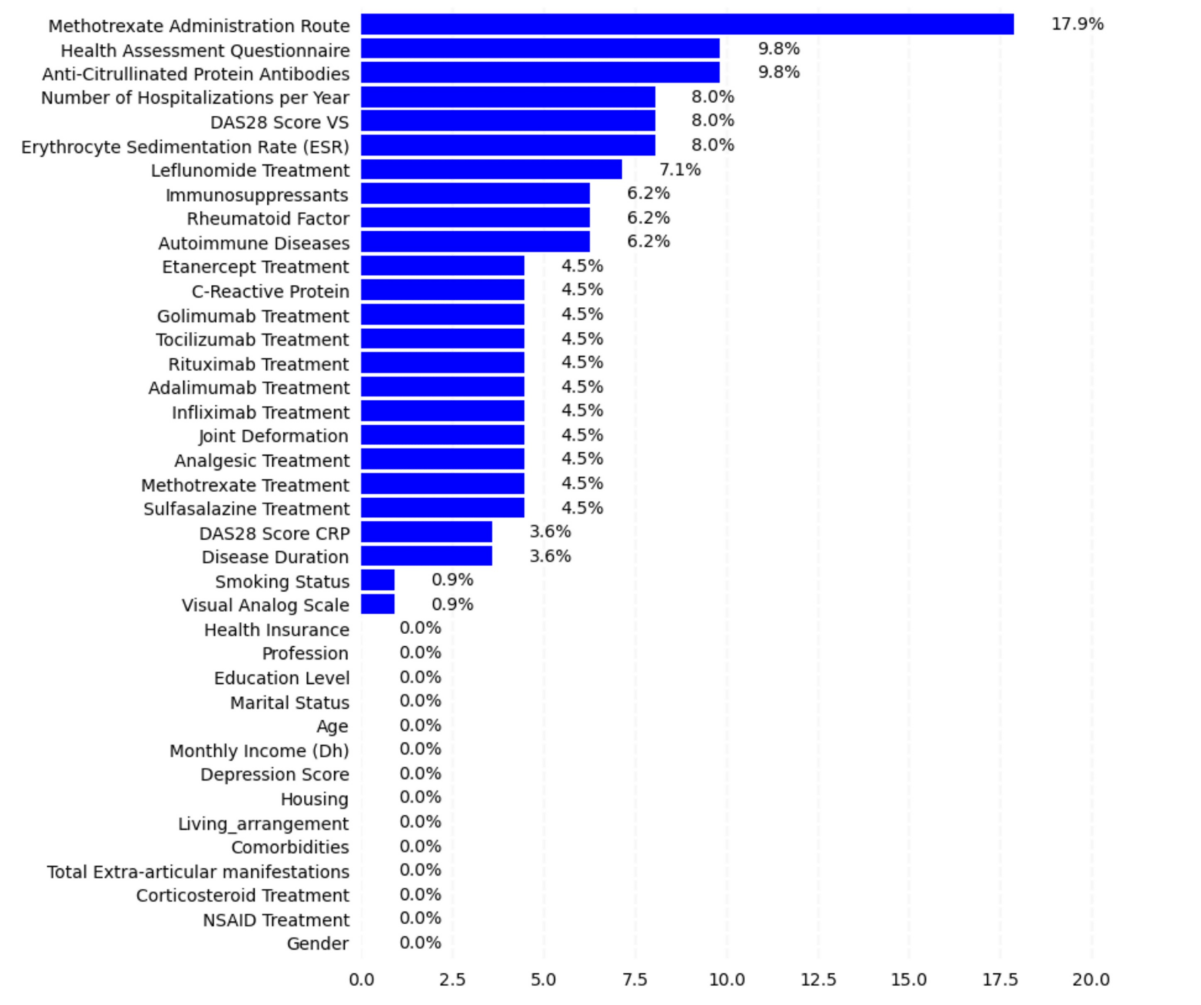
Rate of missing values for each variable in our database The figure illustrates the proportion of missing values for each variable in the dataset. The X-axis represents the variables, and the Y-axis represents the rate of missing values in percentage (%). The data is presented to highlight the extent of missing data across different variables, which is crucial for data preprocessing and analysis

The variable "method of methotrexate intake" displayed the highest rate of missing data, reaching 17.9%. The "Health Assessment Questionnaire (HAQ) score" and "anti-citrullinated peptide antibodies (ACPA)" variables had similar rates of missing data, both at 9.8%. To visualize these percentages, refer to Figure 1, which illustrates the proportion of missing data for each variable.

Feature Selection

**Figure 2:**
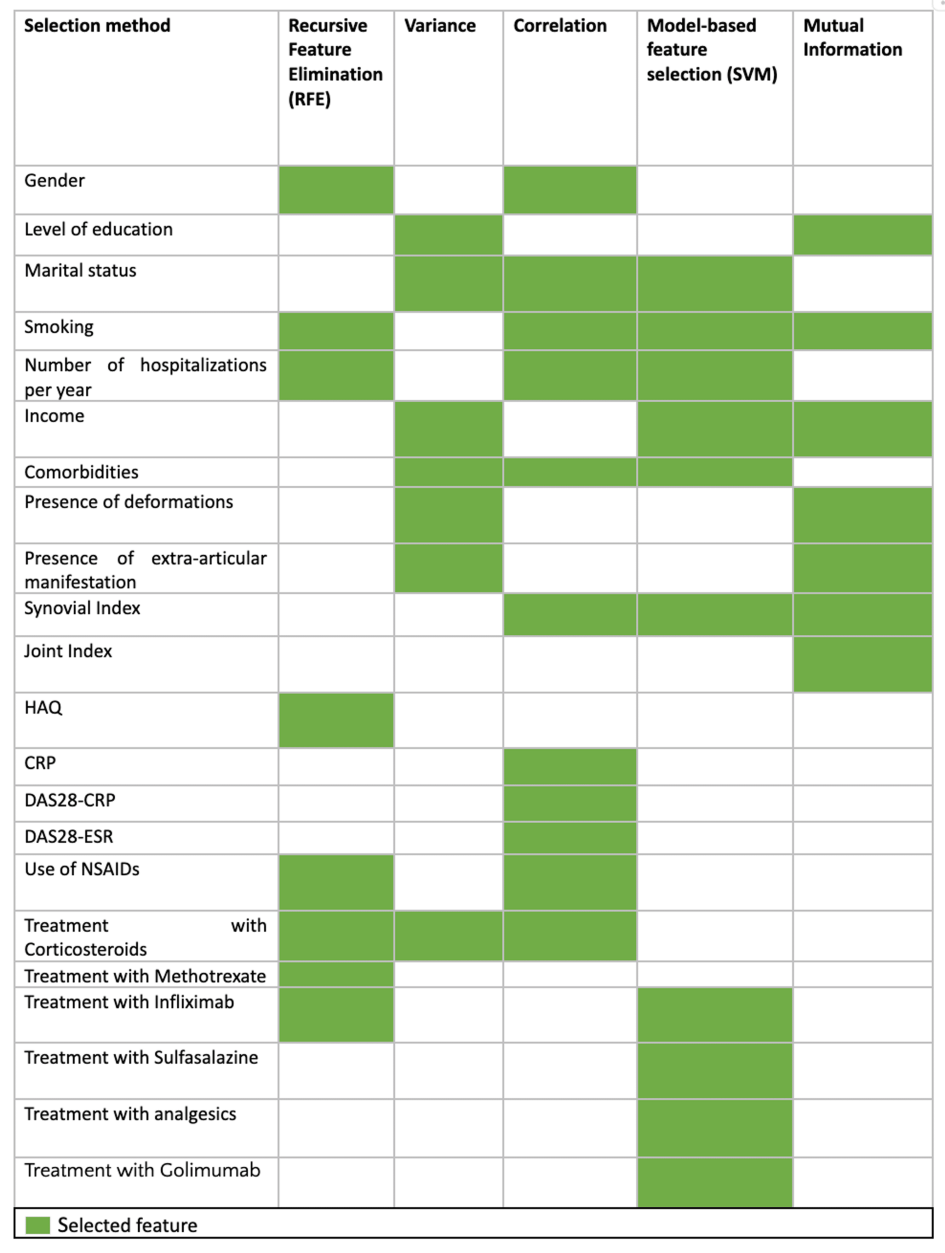
Selected features by each feature selection method This heatmap represents the features selected by different feature selection methods, including recursive feature elimination (RFE), variance threshold, correlation-based selection, model-based selection using support vector machine (SVM), and mutual information. Green cells indicate that the corresponding feature was selected by the respective method

Figure 2 presents the different features selected by each of the methods. This step allowed us, through these different selection methods, to identify the most important features for depression prediction.

Choosing the Best Feature Set

The subset of features selected by the method based on the LR model achieved the best results, with an accuracy of 64.7%. It also demonstrated a precision of 61.1% in predicting positive samples and a recall of 68.7%, indicating its strong ability to identify true positive samples. The model displayed outstanding performance with an F1 score of 64.7%, confirming its high capacity to correctly classify samples, achieving an ROC AUC score of 64.9%. In comparison, the recursive feature elimination (RFE) method showed lower performance, with an accuracy of 50%, precision of 47.4%, recall of 56.2%, F1 score of 51.4%, and ROC AUC score of 50.3%. The detailed results are presented in Table 3.

**Table 3:** Summary of feature selection method performances and SVM model The table presents the performance metrics (Accuracy, Precision, Recall, F1 Score, and ROC AUC Score) for various feature selection methods. All values are numerical RFE: recursive feature elimination; ROC AUC: area under the receiver operating characteristic curve; SVM: support vector machine

Feature selection method	Accuracy	Precision	Recall	F1 score	ROC AUC score
RFE	0.500	0.474	0.562	0.514	0.503
Variance	0.470	0.450	0.562	0.500	0.475
Correlation	0.588	0.545	0.750	0.631	0.597
Logistic regression model	0.647	0.611	0.687	0.647	0.649
Mutuelle	0.529	0.500	0.625	0.555	0.534

Based on these results, we will use the features selected by the method based on the LR model.

Models' Evaluation

The performance of the models evaluated is summarized in Table 4. LR achieved the highest accuracy (76.5%), precision (72.2%), and recall (81.2%). The RF model demonstrated performance similar to SVM, with an accuracy of 64.7%, precision of 60%, and recall of 75%. However, the gradient boosting classifier (GBC) showed the lowest performance among the evaluated models, with an accuracy of 52.9% and relatively low values for precision, recall, and F1 score. The decision tree (DT) displayed the lowest performance with an accuracy of 47.1% and equivalent values for precision, recall, and F1 score.

**Table 4:** Comparative performance of machine learning models with selected features The table presents the performance metrics (accuracy, precision, recall, F1 score, and AUC) for various machine learning models using selected features. All values are numerical and represent the performance of each model AUC: area under the curve

Model	Accuracy	Precision	Recall	F1 score	AUC
Logistic regression	0.765	0.722	0.812	0.765	0.767
Support vector machine	0.647	0.611	0.688	0.647	0.649
Decision tree	0.471	0.438	0.438	0.438	0.469
Random forest	0.647	0.600	0.750	0.667	0.653
Gradient boosting classifier	0.529	0.500	0.438	0.467	0.524

**Figure 3:**
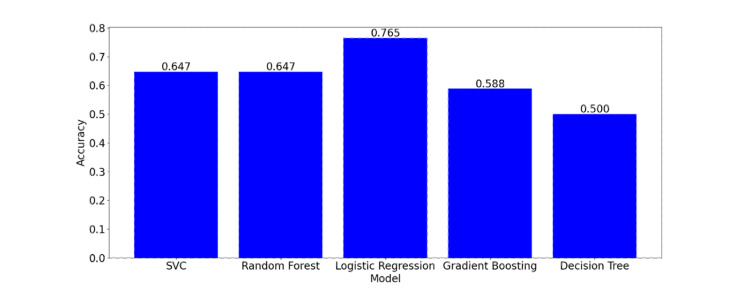
Accuracy of different models The accuracy values are represented as single numeric values SVC: support vector classifier

Figure 3 illustrates the accuracy of different models, providing a visual comparison of their performance. The LR model achieved the highest accuracy at 76.5%.

**Figure 4:**
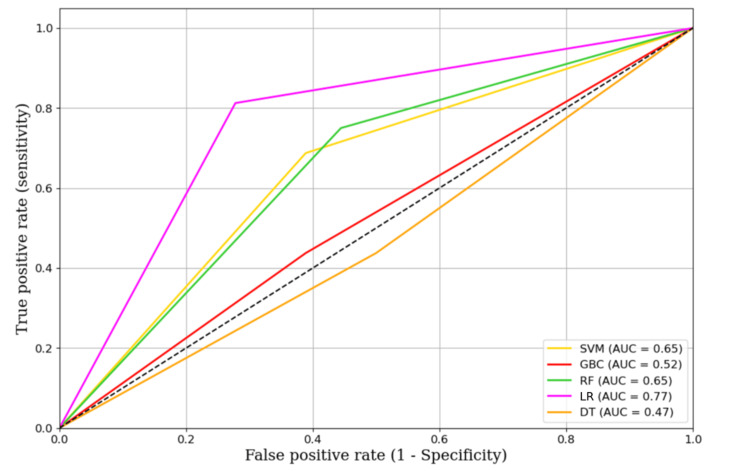
ROCs of the different machine learning models The ROCs illustrate the performance of different machine learning models in terms of sensitivity (true positive rate) versus 1-specificity (false positive rate). The AUC values are provided for each model AUC: area under the curve; DT: decision tree; GBC: gradient boosting classifier; LR: logistic regression; RF: random forest; SVM: support vector machine

Figure 4 presents the ROC curves for the different ML models, illustrating their performance in terms of true positive rates (sensitivity) and false positive rates. The AUC values provide a quantitative measure of each model's effectiveness. LR achieved the highest AUC of 0.77, indicating superior performance in distinguishing between the classes. The SVM and RF models showed moderate performance with AUC values of 0.65 and 0.62, respectively. The GBC had an AUC of 0.56, while the DT model performed the least effectively with an AUC of 0.47. 

## Discussion

The objective of our study was to develop an ML model to detect depression in patients with RA. To the best of our knowledge, our work is the first of its kind, and the literature shows that there is no existing predictive model for depression in RA. Previous studies have predominantly focused on identifying risk factors for depression in RA through traditional statistical analyses [6-7], but none have leveraged ML to build predictive tools. This positions our study as a novel contribution to both rheumatology and computational mental health research. Another strength of our study is the use of a balanced dataset, i.e., we have an equal number of positive and negative samples for depression in RA patients, which enables us to better assess the performance of our ML model and provide more reliable results [21-22].

Our use of five feature selection methods - RFE, variance-based selection, correlation-based selection, model-based logistic regression, and mutual information - aligns with best practices in medical ML, where multi-method approaches are recommended to ensure robustness [23]. For instance, RFE has been widely adopted in depression prediction models for other chronic diseases, such as diabetes, where it improved model interpretability by isolating key predictors like HbA1c levels and social support scores [24]. Similarly, mutual information-based selection has proven effective in psychiatric ML studies for capturing non-linear relationships between biomarkers and depressive symptoms [25]. Our findings corroborate these approaches, as combining multiple selection strategies helped identify a parsimonious yet clinically relevant feature set.

The features selected in our model (e.g., female gender, disease duration, pain intensity, and DAS28 scores) are consistent with prior observational studies. Covic et al. identified female gender and functional disability as strong predictors of depression in RA using LR [26]. Enginar et al. found that higher disease activity (DAS28) and functional impairment (HAQ) were significantly associated with depression in RA patients, consistent with the broader link between disease severity and mental health outcomes [27]. Notably, our model also highlighted socioeconomic status and fatigue - features increasingly recognized in recent literature as critical psychosocial determinants of depression in chronic inflammatory diseases [28]. This convergence with existing evidence underscores the validity of our feature selection process. Female gender and socioeconomic factors, including employment status, are particularly important in the context of RA. In Morocco, women often face additional caregiving responsibilities and social constraints that heighten psychological distress. Moreover, unemployment or job instability can worsen financial stress and social isolation, further increasing the risk of depression.

Our LR model achieved an accuracy of 76.5%, a precision of 72.2%, and a recall of 81.2%. While direct comparisons are limited due to the absence of prior ML models for depression in RA, these metrics are competitive with ML models predicting depression in other chronic conditions. For example, a 2023 study used SVM to predict depression in diabetes patients, achieving 78% accuracy but with a lower recall of 74% [24]. The strong performance of LR in our study aligns with its established role in medical ML, where interpretability is prioritized over marginal gains in accuracy from "black-box" models [29]. 

Our balanced dataset (50% depression prevalence) addresses a key limitation in prior ML studies on mental health, where imbalanced data (typically <20% depression cases) often inflate accuracy metrics [21]. By contrast, our balanced approach, combined with rigorous cross-validation, provides a more realistic assessment of model generalizability - a practice increasingly advocated in computational psychiatry [19]. However, our model’s moderate precision (72.2%) suggests room for improvement compared to biomarker-driven approaches. Recent studies integrating neuroimaging data (e.g., fMRI-based amygdala reactivity) or genetic markers (e.g., serotonin transporter polymorphisms) have achieved >80% precision in depression prediction [30,31]. 

However, it is important to acknowledge the limitations of our study. Firstly, the sample size, though balanced, may still have been insufficient to fully capture the heterogeneity of RA patients, potentially affecting the generalizability of our model. Second, the reliance on self-reported questionnaires and clinical measurements introduces the possibility of subjectivity bias or incomplete data, which could influence the model's performance. Future studies should aim to address these limitations by incorporating larger, more diverse datasets and leveraging objective biomarkers to complement self-reported measures.

Despite these limitations, our findings provide a strong foundation for future research and practical applications. The next phase of our work will focus on integrating the ML model into an e-framework mobile application designed to assist healthcare professionals in the early detection of depression in RA patients. This application will serve as a valuable tool for clinicians, enabling real-time predictions and personalized treatment recommendations during routine consultations. By facilitating timely interventions, this tool has the potential to significantly improve the mental health outcomes of RA patients.

## Conclusions

Our study successfully developed an ML model using LR to predict depression in patients with RA. Among the evaluated algorithms, our model demonstrated the highest accuracy and AUC values, underscoring its reliability in identifying individuals at risk. The integration of this predictive tool into clinical practice could significantly improve the management of RA patients by enabling early detection of depression, thereby facilitating timely and targeted psychological interventions. By leveraging this model, healthcare providers can deliver more personalized care, ultimately enhancing mental health outcomes and overall quality of life for RA patients. Future research should focus on validating the model across diverse populations and incorporating additional predictive features to further enhance its performance.
